# Neuroprotective Effects of Phenolic Constituents from Drynariae Rhizoma

**DOI:** 10.3390/ph17081061

**Published:** 2024-08-13

**Authors:** Jin Sung Ahn, Chung Hyeon Lee, Xiang-Qian Liu, Kwang Woo Hwang, Mi Hyune Oh, So-Young Park, Wan Kyunn Whang

**Affiliations:** 1 College of Pharmacy, Chung-Ang University, Seoul 06974, Republic of Korea; jsahn0717@gmail.com (J.S.A.);; 2 College of Pharmacy, Dankook University, Cheonan 31116, Republic of Korea; 3 School of Pharmacy, Hunan University of Chinese Medicine, Changsha 410208, China

**Keywords:** *Drynaria fortunei* J. Smith, amyloid beta, cholinesterase, BACE1, MAO-B, sAPPβ, β-secretase, Th T assay, Alzheimer’s disease

## Abstract

This study aimed to provide scientific data on the anti-Alzheimer’s disease (AD) effects of phenolic compounds from Drynariae Rhizoma (DR) extract using a multi-component approach. Screening of DR extracts, fractions, and the ten phenolic compounds isolated from DR against the key AD-related enzymes acetylcholinesterase (AChE), butyrylcholinesterase (BChE), β-site amyloid precursor protein cleaving enzyme 1 (BACE1), and monoamine oxidase-B (MAO-B) confirmed their significant inhibitory activities. The DR extract was confirmed to have BACE1-inhibitory activity, and the ethyl acetate and butanol fractions were found to inhibit all AD-related enzymes, including BACE1, AChE, BChE, and MAO-B. Among the isolated phenolic compounds, compounds (**2**) caffeic acid 4-O-β-D-glucopyranoside, (**6**) kaempferol 3-O-rhamnoside 7-O-glucoside, (**7**) kaempferol 3-o-b-d-glucopyranoside-7-o-a-L-arabinofuranoside, (**8**) neoeriocitrin, (**9**) naringin, and (**10**) hesperidin significantly suppressed AD-related enzymes. Notably, compounds **2** and **8** reduced soluble Amyloid Precursor Protein β (sAPPβ) and β-secretase expression by over 45% at a concentration of 1.0 μM. In the thioflavin T assay, compounds **6** and **7** decreased Aβ aggregation by approximately 40% and 80%, respectively, and degraded preformed Aβ aggregates. This study provides robust evidence regarding the potential of DR as a natural therapeutic agent for AD, highlighting specific compounds that may contribute to its efficacy.

## 1. Introduction

Drynariae Rhizoma (DR) belongs to the Polypodiaceae family and is registered in the Chinese Pharmacopoeia and the Korean Pharmacopoeia as *Drynaria fortunei* J. Smith [1,2]. DR is recorded in the Illustrated Chinese Materia Medica (Chinese bonchodogam) as being distributed south of the Yangtze River in China [3], and it is currently widely cultivated and distributed in Asian countries such as Japan and Korea [4]. DR has been reported to be rich in phenolic components with excellent physiological activities, such as naringin [5], neoeriocitrin [6,7,8], and caffeic acid 4-O-β-d-glucoside [6]. Additionally, in a study analyzing the principal components of DR, the contents of naringin, neoeriocitrin, 5,7-dihydroxychromone-7-O-neohesperidoside, and caffeic acid 4-O-β-d-glucoside were reported to be 0.93–9.86 mg/g, 0.74–7.59 mg/g, 0.05–2.48 mg/g, and 0.27–2.51 mg/g, respectively. These findings indicate that DR contains rich phenolic components [9]. DR has been widely used for thousands of years as a natural drug for bones and joints [10], including for osteoblast proliferation [11], osteocyte activation [12], and anti-osteoporosis [13]. It is one of the most frequently used traditional medicines, known to replenish the kidney, strengthen the bones, promote the healing of fractures, and relieve pain [14]. Recent studies have continued to explore the various effects of DR. The water extract of DR was found to increase MMP-2 activity and activate VEGF/VEGFR expression, thereby promoting cell migration, angiogenesis, and wound healing [15]. Total flavonoids of DR were demonstrated to prevent intervertebral disc degeneration in mice by inhibiting inflammation and matrix degradation [16]. Additionally, DR extract reduced body weight and lipid accumulation in high-fat diet-induced obese mice while strengthening bones and regulating obesity-related gene expression [17]. Thus, research on the various effects of DR has been actively conducted until recently. In particular, attention has been paid to its potential in treating neurodegenerative diseases such as Alzheimer’s disease (AD) [6,18].

Alzheimer’s disease (AD), a progressive and irreversible neurodegenerative condition, is the leading cause of dementia among the elderly. AD leads to the gradual decline of cognitive function, memory loss, and changes in behavior while also encompassing symptoms such as paranoid disorders, delusions, and a lack of social appropriateness, ultimately leading to death [19,20,21,22,23]. Multiple factors are involved in AD progression. Several hypotheses proposed include the cholinergic hypothesis, the amyloid beta (Aβ) hypothesis, the oxidative stress hypothesis, and the inflammatory hypothesis [24]. The cholinergic hypothesis served as the foundation for developing synaptic treatments aimed at preserving the function of the remaining cholinergic system. Biomarkers associated with cholinergic neurons, such as acetylcholinesterase (AChE) and butyrylcholinesterase (BChE), play crucial roles in the synthesis and breakdown of acetylcholine (ACh) and butyrylcholine (BCh), respectively [25]. Monoamine oxidase-B (MAO-B) has recently emerged as a potential therapeutic target for AD because of its association with aberrant γ-aminobutyric acid production in reactive astrocytes [26]. The amyloid hypothesis posits that Aβ peptides (38–43 amino acids long) are byproducts of the cleavage of amyloid precursor protein (APP). This cleavage involves key enzymes, namely β-secretase, also referred to as β-site amyloid precursor protein cleaving enzyme 1 (BACE1), and γ-secretase [27,28,29]. β-secretase cleaves the ectodomain of APP, producing two fragments: soluble Amyloid Precursor Protein β (sAPPβ) and C-terminal fragment β (CTFβ) [30,31,32]. sAPPβ is released from the cell, while CTFβ remains and is further cleaved by γ-secretase to generate the 4 kDa Aβ peptide [33,34,35]. The Aβ peptides aggregate into oligomers, fibrils, and amyloid plaques, leading to impaired synaptic function and brain damage [36]. Hence, strategies aimed at diminishing the production of neurotoxic Aβ and preventing its aggregation present promising avenues for therapeutic intervention and disease prevention in AD [37]. Recent studies have explored the potential of DR in treating AD. In 2005, Dr. Yang reported that DR enhanced cognitive abilities [38]. In 2009, we investigated natural neuroprotective substances that inhibit Aβ-induced toxicity using PC12 cells. Among 400 medicinal herbs, the methanol extract of DR exhibited a promising neuroprotective effect, with an ED50 value of less than 10 μg/mL. This suggests that DR is a promising potential treatment as a natural resource for Alzheimer’s disease (AD) [39]. In 2015, Yang et al. reported that an aqueous extract of DR reversed Aβ25–35-induced axonal atrophy in cultured cortical neurons of mice. Notably, the phenolic compounds isolated from the extract (2S)-neoeriocitrin (1 and 10 μM) and caffeic acid 4-O-glucoside (10 μM) showed significant axonal regrowth effects on Aβ25–35-induced atrophy [6]. In 2017, Yang et al. demonstrated that DR can improve AD-like pathology and memory deficits in 5XFAD model mice. In particular, naringenin, a phenolic compound isolated from DR, and its metabolite, naringenin-7-O-glucuronide, can cross the blood–brain barrier and exhibit significant neuroprotective effects against Aβ-induced axonal atrophy. These findings highlight the potential of DR as a treatment for AD and emphasize the importance of metabolite identification in understanding the therapeutic mechanisms of natural compounds [40]. Additionally, it has been reported that *Drynaria quercifolia*, from the same Polypodiaceae family as DR, ameliorates scopolamine-induced memory impairment in mice due to its anticholinesterase and antioxidant activities [41]. Previous reports on the anti-AD effects of DR have primarily focused on the neuroprotective properties of DR extracts, fractions, or specific compounds. However, there is a notable lack of scientific data supporting pharmacological studies that screen various secondary metabolites of DR and evaluate their anti-AD activities. In particular, no studies have investigated their effects on Aβ formation and aggregation or identified the active ingredients responsible. In this study, we utilized extracts and fractions of DR, along with ten phenolic compounds isolated from DR, to evaluate various anti-AD activities. This was carried out through enzyme activity assays for AChE, BChE, BACE1, and MAO-B. We conducted an in-depth analysis of the inhibitory activity on Aβ formation and aggregation as well as the disintegration effect on preformed Aβ aggregates for selected compounds. Through this study, we aimed to confirm the potential efficacy of phenolic compounds from DR extract as a natural AD treatment through various activities and provide scientific data to support pharmacological research.

## 2. Results and Discussion

### 2.1. Inhibitory Activity of DR Extract and Fractions on AD-Related Enzymes

We investigated the inhibitory activity of DR extract and its fractions on acetylcholinesterase (AChE), butyrylcholinesterase (BChE), β-site APP-cleaving enzyme 1 (BACE1), and monoamine oxidase-B (MAO-B) assay (Table 1). The DR extract did not exhibit significant inhibitory activities against AChE, BChE, and MAO-B. However, ethyl acetate (EtOAc) and *n*-butanol (BuOH) fractions inhibited AChE with IC_50_ values of 618.33 ± 1.75 μg/mL and 607.73 ± 2.07 μg/mL, respectively, and BChE with IC_50_ values of 391.70 ± 3.12 μg/mL and 439.14 ± 3.12 μg/mL, respectively. In the MAO-B analysis, the IC_50_ was 75.22 ± 1.75 μg/mL for EtOAc fraction. The AChE-, BChE-, and MAO-B-inhibitory effects of the DR extract were difficult to confirm, but the BACE1-inhibitory activity was confirmed with an IC50 of 207.07 ± 5.14 μg/mL. In addition, the EtOAc and BuOH fractions also showed BACE1 inhibition with IC50 values of 85.36 ± 3.24 μg/mL and 97.82 ± 4.11 μg/mL, respectively. Therefore, these results suggest that the DR extract, along with the EtOAc and BuOH fractions, significantly affects Aβ production-inhibitory activity.

### 2.2. Inhibitory Activity of Ten DR Compounds on AD Enzymes

**Figure 1:**
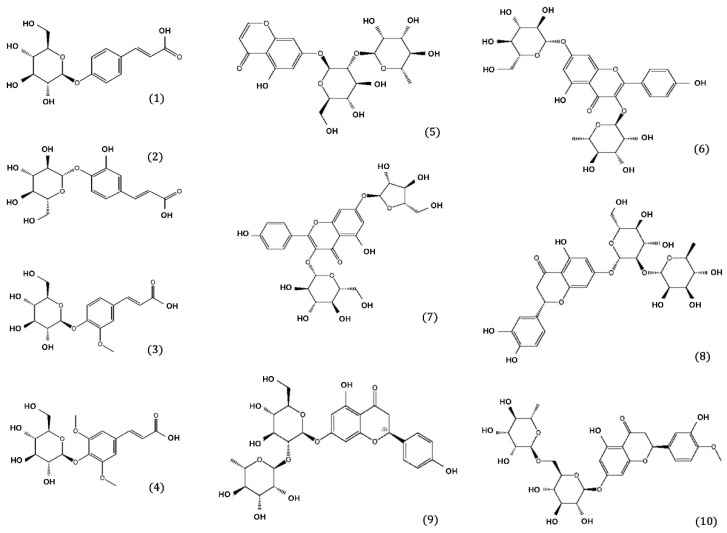
Chemical structures of ten compounds isolated from *Drynaria fortunei*.

We focused on EtOAc and BuOH fractions, which were confirmed to be effective in treating AD through AD-related enzyme activity tests of DR extracts and fractions. The multi-component AD-related enzyme screening analysis of DR was performed on ten phenolic compounds isolated from these fractions. The ten phenolic compounds used were isolated in a 2023 study from EtOAc and BuOH fractions of DR [42]. They were separated, purified, and validated through bioassay-guided fractionation using ODS, Sephadex LH-20, and MCI open-column chromatography. The exact compounds were (**1**) coumaric acid 4-O-β-D-glucopyranoside, (**2**) caffeic acid 4-O-β-D-glucopyranoside, (**3**) lavandoside, (**4**) trans-p-sinapoyl-β-D-glucopyranoside, (**5**) 5,7-dihydroxychromone-7-O-neohesperidoside, (**6**) kaempferol 3-O-rhamnoside 7-O-glucoside, (**7**) kaempferol 3-o-b-d-glucopyranoside-7-o-a-L-arabinofuranoside, (**8**) neoeriocitrin, (**9**) naringin, and (**10**) hesperidin. Figure 1 shows the chemical structures of the ten compounds analyzed.

The ten compounds were screened for their anti-AD activity using ChE (AChE and BChE), BACE1, and MAO-B analyses. The results, shown in Table 2, indicate that some compounds exhibited concentration-dependent and consistent inhibitory activity. (Table 2).

After confirming the cholinesterase-inhibitory activity of ten compounds, the highest AChE-inhibitory activity was observed for compounds **2**, **8**, **1**, **7**, and **10**, with IC_50_ values of 14.77 ± 0.07, 31.65 ± 0.14, 33.39 ± 0.42, 47.33 ± 0.22, and 57.70 ± 5.96 μM, respectively. For BChE-inhibitory activity, compounds **7**, **8**, **4**, **6**, **5**, and **1** exhibited IC_50_ values of 28.83 ± 0.31, 30.04 ± 0.23, 30.63 ± 0.38, 32.61 ± 5.66, 38.76 ± 0.71, and 53.78 ± 1.15 μM, respectively. Compound **3** did not demonstrate cholinesterase-inhibitory activity. Compounds **1**, **7**, and **8** showed excellent activity in both AChE and BChE, with compound **2** displaying the most notable AChE-inhibitory activity at 14.77 ± 0.07 μM. Regarding BACE1-inhibitory activity, compound **8** (IC_50_ 13.66 ± 0.14 μM) exhibited effects comparable to the positive control (quercetin, IC_50_ 9.72 ± 3.72 μM). Additionally, compounds **7**, **2**, **10**, **9**, and **6** showed significant activity with IC_50_ values of 39.11 ± 0.17, 44.98 ± 0.48, 55.72 ± 0.17, 64.95 ± 0.17, and 83.27 ± 0.33 μM, respectively. Regarding MAO-B-inhibitory activity, compound **8** displayed high activity (IC_50_ 2.64 ± 0.016 μM), while compounds **9** and **7** showed significant inhibitory activity with IC_50_ values of 51.26 ± 0.14 and 65.84 ± 3.72 μM, respectively.

The inhibitory activity of ten compounds isolated from DR against AD enzymes was evaluated through an AD activity screening. Hydroxycinnamic acid glycoside compounds (compounds **1**, **2**, **3**, and **4**) and chromone glycoside compound **5** demonstrated some inhibitory effects on AChE, BChE, BACE1, and MAO-B. However, some results showed IC_50_ values exceeding 1000 μg/mL or were non-detectable.

Flavonoid glycoside compounds **6**, **7**, **8**, **9**, and **10** showed significant effectiveness against all AD-related enzymes. Additionally, as compound **2** had high Aβ-inhibitory activity in previous studies [6], this study confirmed its excellent BACE1- and AChE-inhibitory activity. Based on these results, six phenolic compounds (compounds **2**, **6**, **7**, **8**, **9**, and **10**) were selected for in-depth analysis. These compounds were evaluated for their ability to inhibit Aβ production by reducing β-secretase expression in Amyloid Precursor Protein-expressing Chinese Hamster Ovary (APP-CHO) cells. The decomposition of and reduction in Aβ aggregates were assessed through thioflavin T (Th T) analysis.

### 2.3. Inhibitory Activity of Six Phenolic DR Compounds on Aβ Production

**Figure 2:**
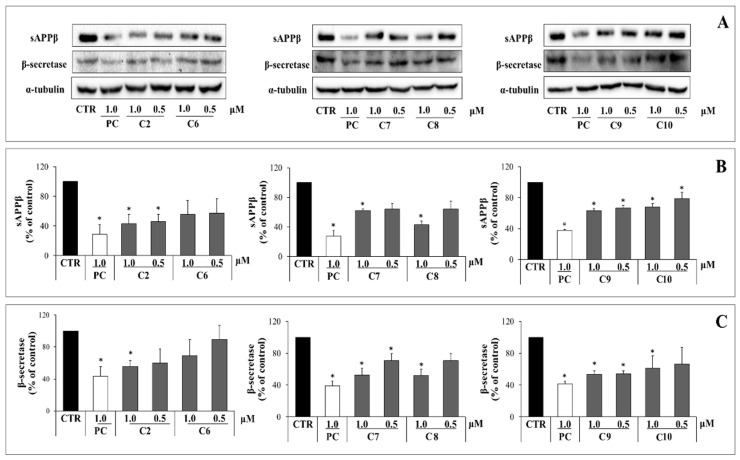
The effects of six phenolic compounds (compounds **2**, **6**, **7**, **8**, **9**, and **10**) on sAPPβ and β-secretase production. (**A**) sAPPβ and β-secretase levels in APP-CHO cells treated with different concentrations (1.0 and 0.5 μM) of six compounds were determined by Western blot analysis. (**B**,**C**) Graphs show sAPPβ (**B**) and β-secretase (**C**) levels compared to DMSO-treated controls. Values are expressed as a percentage of DMSO-treated control. All data are presented as the mean ± standard deviation of three different experiments. * *p* < 0.05: significant difference from the DMSO-treated control group.

We conducted screening for anti-AD enzyme activity of ten compounds isolated from DR and selected six phenolic compounds (compounds **2**, **6**, **7**, **8**, **9**, and **10**) for analysis of their Aβ production inhibition. APP is cleaved by β-secretase to generate sAPPβ and a C-terminal fragment, which is then cleaved by γ-secretase to produce Aβ. In turn, Aβ accumulation is a key feature of AD [35,43,44]. We used Western blot analysis to measure sAPPβ and β-secretase levels in APP-CHO cells to evaluate the inhibitory effect of six phenolic compounds on Aβ production. The whole Western blot results, showing the effects of the six phenolic compounds on APP-CHO cells, are provided as Figure S1. The potential cytotoxicity of these phenolic compounds was evaluated using an MTT assay on the APP-CHO cell line. The APP-CHO cell survival rate for all six phenolic compounds was >90% at concentrations of 1.0 μM and 0.5 μM (Figure S2). Their sAPPβ- and β-secretase-inhibitory activities were evaluated at non-cytotoxic concentrations. The six phenolic compounds decreased sAPPβ and β-secretase expression levels in a concentration-dependent manner (Figure 2). In the case of sAPPβ, all six compounds reduced sAPPβ levels by 30% compared to dimethyl sulfoxide (DMSO)-treated controls at a concentration of 1.0 μM. Specifically, compounds **2** and **8** reduced sAPPβ levels by >50% at a concentration of 1.0 μM. Regarding β-secretase, all six compounds reduced β-secretase levels to 30% of DMSO-treated controls at a concentration of 1.0 μM. Compounds **7**, **8**, and **9** reduced β-secretase levels by 40% at a concentration of 1.0 μM. Specifically, compound **8** reduced sAPPβ and β-secretase expression levels by 55% and 45%, respectively, at a concentration of 1.0 μM. Additionally, compound **2** reduced sAPPβ and β-secretase expression levels by 50% and 40%, respectively, even at a low concentration of 0.5 μM. Thus, compounds **2**, **6**, **7**, **8**, **9**, and **10** all inhibit Aβ production. Particularly, compounds **2** and **8** showed relatively high efficacy in inhibiting sAPPβ and β-secretase expression.

### 2.4. Analysis of Aβ Aggregation Reduction and Preformed Aβ Aggregate Degradation Caused by Six Phenolic DR Compounds

**Figure 3:**
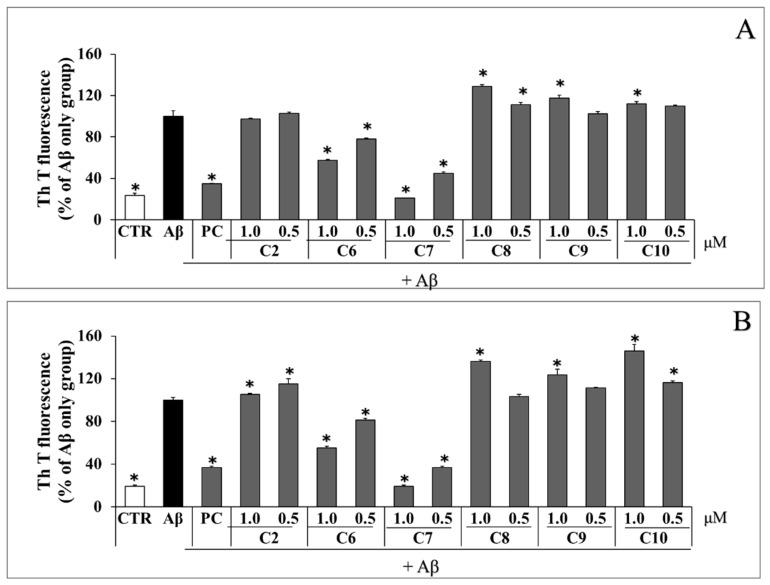
Inhibition of Aβ aggregation and degradation of preformed Aβ aggregates by six phenolic compounds (compounds **2**, **6**, **7**, **8**, **9**, and **10**). (**A**) Aβ was incubated with six phenolic compounds at concentrations of 50 μM and 10 μM. After 24 h, Aβ aggregation was assessed using the Th T assay. (**B**) Aβ pre-aggregated for 24 h was exposed to six phenolic compounds at concentrations of 1.0 and 0.5 μM. After another 24 h, Aβ disaggregation was evaluated using the Th T assay. All data are presented as the mean ± standard deviation of three independent experiments. * *p* < 0.05: significant difference from the Aβ-only group.

The effect of six phenolic compounds on Aβ aggregation and degradation of preformed Aβ aggregates was analyzed using the Th T assay (Figure 3). Compounds **6** and **7** significantly reduced Aβ aggregation compared to the DMSO-treated Aβ-alone control group (Figure 3A). Specifically, compound **6** decreased Aβ aggregation by about 40% at a concentration of 1.0 μM relative to the DMSO-treated control. Additionally, compound **7** reduced Aβ aggregation to approximately 80% and 55% of the levels observed in the DMSO-treated control at concentrations of 1.0 μM and 0.5 μM, respectively. Pre-aggregated Aβ was incubated with the compounds at concentrations of 1.0 μM and 0.5 μM for 24 h. Then, the extent of Aβ aggregation was measured using Th T analysis. Compounds **6** and **7** increased Aβ degradation compared to the DMSO-treated Aβ-alone control group (Figure 3B). Compound **6** increased Aβ degradation to approximately 45% of that in the DMSO-treated control at 1.0 μM. Furthermore, compound **7** increased Aβ degradation to about 80% and 60% of that in the DMSO-treated control at concentrations of 1.0 μM and 0.5 μM, respectively. Therefore, compounds **6** and **7** demonstrated excellent efficacy in reducing Aβ aggregation and degrading preformed Aβ aggregates.

## 3. Materials and Methods

### 3.1. Samples Derived from Drynariae Rhizoma (DR): Extracts, Fractions, and Compounds

The DR (*Drynaria fortunei* J. Smith) samples utilized in this study were obtained from a previous investigation [42]. The samples included an 80% methanol extract of DR, its fractions (dichloromethane, ethyl acetate, butanol, and water), and ten compounds isolated through a bioassay-guided method using ethyl acetate and butanol fractions.

### 3.2. Equipment and Reagents

The AChE- and BChE-inhibitory, MTT, and Th T assays were analyzed using a UV–visible (VIS) spectrophotometer (Biotek, EPOCH2, Winooski, VT, USA), ensuring accurate measurements. For BACE1- and MAO-B-inhibitory assays, a multimode microplate reader (SpectraMax M3, Molecular Devices, San Jose, CA, USA) was employed, providing precise readings. Analytical-grade DMSO from DEAJUNG Chemical, Korea, served as a solvent in the bioassays. Reagents and solvents, including AChE from electric eel, acetylthiocholine iodide (ATCh), BChE from equine serum, S-butyrylthiocholine iodide (BTCh), 5,5′-dithiobis [2-nitrobenzoic acid] (DTNB), hesperidin, berberine, and quercetin, were obtained from Sigma-Aldrich Co., St. Louis, USA, ensuring reliability. Additionally, the BACE1-inhibitory assay utilized the BACE1 (β-secretase) FRET assay kit from PanVera^®^ Corporation, Madison, USA, while the MAO-B-inhibitory assay employed the MAO-B Inhibitor Screening Kit (Fluorometric) ab284511 from Abcam plc Co., Cambridge, UK.

MTT analysis was evaluated using Biosesang, Yongin-si, Korea. RPMI 1640 medium (Welgene, Gyeongsan-si, Republic of Korea), fetal bovine serum (FBS; Gibco, Grand Island, NY, USA), and geneticin (Thermo Fisher Scientific, Waltham, MA, USA) were used for cell culture. The signal intensity of Western blots was quantified using Bio-Rad software (version 5.2.1., Bio-Rad, Hercules, CA, USA). Trans-Blot^®^ TurboTM (Bio-Rad, Hercules, CA, USA) facilitated Western blot analysis. Primary antibodies including sAPPβ (Immuno-Biological Laboratories, Fujioka, Japan) and α-tubulin (Sigma Aldrich, Saint Louis, MO, USA) were utilized. Horseradish peroxidase (HRP; Bio-Rad, Hercules, CA, USA) and ECL solution reagent (Advansta, Menlo Park, CA, USA) were used for protein visualization, which was conducted using the ChemiDocTM XRS+ (Bio-Rad, Hercules, CA, USA). Aβ1-42 used for the Th T assay was purchased from GL Biochem, Shanghai, China. The Th T was obtained from Sigma Aldrich, ensuring consistency and reliability in the experimental procedures. The BACE1 (β-secretase) FRET assay kit was purchased from Pan Vera Co. (Madison, WI, USA), and the MAO-B inhibitor screening kit was purchased from ab284511 (Abcam plc, Cambridge, UK). Other chemicals and solvents used were of analytical grade.

### 3.3. Cholinesterase (ChE)-Inhibitory Assay

The inhibitory activities against cholinesterases (AChE and BChE) were evaluated using a spectrophotometric method previously developed by Ellman et al. [45]. Specifically, ATCh and BTCh were used as substrates to measure the inhibitory effects on AChE and BChE, respectively. The assay mixture consisted of 140 μL of 0.1 M sodium phosphate buffer at pH 7.8, 20 μL of a 0.38 U/mL solution of either AChE or BChE, and 20 μL of the test sample solution. This mixture was thoroughly mixed and then incubated at room temperature for 15 min. All test samples and positive control (berberine) were dissolved in 10% analytical grade DMSO at five different final concentrations (ranging from 10 to 500 μg/mL for extracts and fractions or 10 to 500 μM for isolated compounds). Reactions were initiated by adding 10 μL of 0.5 mM DTNB and 10 μL of either 0.6 mM ATCh or BTCh. ChE-inhibitory activity was monitored by measuring the formation of the yellow 5-thio-2-nitrobenzoate anion at 412 nm over 15 min (Epoch, BioTek, Winooski, VT, USA), resulting from the reaction of DTNB and thiocholine released from ATCh or BTCh. All reactions were conducted in 96-well microplates and performed in triplicate. The percentage of inhibition (%) was determined using the following formula: inhibition (%)={(Ac−As)/Ac}×100, where Ac represents the enzyme activity in the absence of the test sample, and As represents the enzyme activity in the presence of the test sample. The ChE-inhibitory activities of each sample were quantified in terms of IC_50_, which was calculated from the log–dose inhibition curve.

### 3.4. β-Site Amyloid Precursor Protein Cleaving Enzyme 1 (BACE1)-Inhibitory Assay

The BACE1 inhibition assay was conducted using the BACE1 (β-secretase) FRET Assay Kit, Red Protocol, part number P2985 (PanVera^®^ Corporation, Madison, WI, USA), following the manufacturer’s recommended protocol with minor modifications. The assay mixture consisted of 10 μL of BACE1 (1.0 U/mL), 10 μL of substrate (750 nM Rh-EVNLDAEFK-Quencher in 50 mM ammonium bicarbonate), the assay buffer (50 mM sodium acetate, pH 4.5), and 10 μL of the test samples. All samples, including the positive control (quercetin), were dissolved in 10% DMSO at five different final concentrations (ranging from 30 to 3000 μg/mL for extracts and fractions or 5 to 100 μM for isolated compounds). The reaction mixture was incubated for 60 min at room temperature in darkness. Before measurement, a stop solution (2.5 M sodium acetate) was added to halt the reaction. BACE1 enzyme activity was assessed by monitoring the proteolysis of two fluorophores (Rh-EVNLDAEFK-Quencher), forming the fluorescent donor (Rh-EVNL) with increased fluorescence wavelengths at 530–545 nm (excitation) and 570–590 nm (emission), respectively. All reactions were recorded in black 96-well microplates and performed in triplicate. The percentage of inhibition (%) was calculated using the following formula: inhibition (%)=[1−(S60−S0)/(C60−C0)]×100, where C60 represents the fluorescence of the control after incubation, C0 represents the initial fluorescence of the control, S60 represents the fluorescence of the tested sample after incubation, and S0 represents the initial fluorescence of the tested sample. The BACE1-inhibitory activities of each sample were quantified in terms of IC_50_, which was determined from the log–dose inhibition curve.

### 3.5. Monoamine Oxidase-B (MAO-B)-Inhibitory Assay

The MAO-B-inhibition assay was conducted using the MAO-B Inhibitor Screening Kit (Fluorometric) ab284511 (Abcam plc, Cambridge, UK), following the manufacturer’s recommended protocol with minor modifications. The enzyme solution (50 µL) and the test sample (10 µL) were adjusted as per the manual instructions. All samples, including the positive control (selegiline), were dissolved in 10% analytical grade DMSO at five final concentrations (ranging from 2.5 to 5000 μg/mL for extracts and fractions or 0.5 to 100 μM for isolated compounds). After mixing, samples and enzymes were incubated at 25 °C for 10 min. Subsequently, 40 μL of MAO-B substrate (comprising MAO-B assay buffer, MAO substrate, developer, and OxiRed probe) was added, followed by further incubation at 25 °C for 5 min. Fluorescence was measured using enzyme-linked immunosorbent assay within 40 min with dynamic fluorescence (Ex/Em = 535/587 nm). All reactions were recorded in black 96-well microplates and performed in triplicate. The slope for all samples, including the enzyme control (EC), was calculated by dividing the net ΔRFU (RFU2–RFU1) values by the time Δt (T2 − T1). The % relative inhibition was computed as follows: % relative inhibition=(slope of EC−slope of S)/slope of EC×100. The MAO-B-inhibitory activities of each sample were quantified in terms of IC_50_, as determined from the log–dose inhibition curve.

### 3.6. Cell Culture and MTT Assay

APP-CHO cells were obtained from the Korean Cell Line Bank (Seoul, Republic of Korea) and cultured in RPMI 1640 medium supplemented with 10% FBS. The cells were incubated in a humidified atmosphere of 5% CO_2_ at 37 °C. APP-CHO cells [46], which are Chinese hamster ovary cells stably expressing APP and established in our lab [37], were also cultured in RPMI 1640 medium. This medium was supplemented with 10% heat-inactivated FBS and geneticin at a concentration of 50 μg/mL. Cell viability was assessed using the MTT assay. Briefly, APP-CHO cells were seeded in a 96-well plate at a density of 2 × 10^4^ cells/100 μL of medium per well and incubated at 37 °C overnight for cell adhesion. Before treatment with test samples, the medium was replaced with FBS-free RPMI 1640 for 1 h. Test samples dissolved in DMSO were added to the wells, followed by further incubation at 37 °C for 24 h. Afterward, 10 μL of 5 mg/mL MTT solution was added to each well and incubated at 37 °C for 3 h. Following medium removal, 100 μL of DMSO was added to dissolve the MTT formazan crystals. Then, the absorbance was measured at 540 nm using an E-max precision microplate reader (Epoch, BioTek, Winooski, VT, USA). Each experiment was performed in triplicate.

### 3.7. Western Blot Analysis

APP-CHO cells were seeded in six-well plates at a density of 6 × 10^5^ cells/1000 μL of medium per well and incubated at 37 °C overnight for cell attachment. Then, the medium was replaced with FBS-free RPMI 1640 for 1 h, followed by treatment with test samples at 37 °C. After 24 h, the cells were washed with PBS and lysed in Laemmli sample buffer. The collected cell lysates were boiled at 100 °C for 10 min. For Western blot analysis, 20 μg of proteins were separated by 7.5% SDS-PAGE and transferred to a PVDF membrane using Trans-Blot^®^ Turbo TM (Bio-Rad, Hercules, CA, USA). The membrane was blocked with 5% skim milk in PBS for 1 h and incubated with primary antibodies overnight at 4 °C. The primary antibodies used were sAPPβ antibody, BACE1 antibody, and α-tubulin antibody diluted at 1:1000, 1:1000, and 1:25,000, respectively. After washing with PBS-T (0.1% Tween 20 in PBS) three times for 10 min each, secondary antibodies, diluted to 1:2500, were incubated for 1 h. Protein detection was performed using ECL solution reagent and protein visualization (ChemiDoc^TM^ XRS+., Bio-Rad, Hercules, CA, USA). The signal intensity of the bands was quantified and normalized to the signal intensity of the α-tubulin band. Results were expressed as a percentage of DMSO-treated control.

### 3.8. Thioflavin T (Th T) Assay

The Th T assay was conducted to assess the aggregation of Aβ into oligomers and fibrils. Aβ1-42 was dissolved in DMSO at a concentration of 1 mg/mL, and test samples were also dissolved in DMSO. Aβ1-42 (20 μM) was incubated with various concentrations of test samples at 37 °C for 24 h to evaluate the effects of test samples on Aβ aggregation. Subsequently, 3 μM of Th T was added, and fluorescence was measured after 30 min using an E-max precision microplate reader with excitation at 442 nm and emission at 485 nm (Epoch, BioTek, Winooski, VT, USA). Aβ treated with DMSO only served as a control, and each assay was performed in triplicate. Then, the Th T assay was performed to assess the effects of test samples on the disaggregation of preformed Aβ aggregates. Briefly, 20 μM of Aβ1-42 was incubated at 37 °C for 24 h. Various concentrations of test samples were then added and incubated at 37 °C for an additional 24 h. Subsequently, 3 μM of Th T was added, and fluorescence was measured after 30 min using an E-max precision microplate reader with excitation at 442 nm and emission at 485 nm (Epoch, BioTek, Winooski, VT, USA). Aβ treated with DMSO only served as a control, and each assay was performed in triplicate.

### 3.9. Statistical Analysis

For statistical analysis, we initially conducted a variance test using Levene’s test. If the resulting *p*-value exceeded 0.05, we accepted the assumption of homoscedasticity. Subsequently, we performed two or more group comparisons using a one-way analysis of variance, followed by Fisher’s least significant difference test (SPSS version 27.0, Armonk, NY, USA). In cases when the *p*-value from Levene’s test was <0.05, indicating heteroscedasticity, we rejected the assumption of homoscedasticity. In such instances, two or more group comparisons were evaluated using the Kruskal–Wallis test and the Mann–Whitney U-test (Jamovi software version 2.3.28). A *p*-value <0.05 (* *p* < 0.05) indicated a statistically significant difference.

## 4. Conclusions

This study explored the anti-AD of DR using a multi-component approach, demonstrating significant results. The inhibitory activities of DR extracts, fractions, and ten phenolic compounds on AD-related enzymes (AChE, BChE, BACE1, and MAO-B) were evaluated. The DR extract showed Aβ-inhibitory activity, with a BACE1 IC_50_ value of 207.07 ± 5.14 μg/mL. Ethyl acetate and butanol fractions had inhibitory activities against AChE, BChE, BACE1, and MAO-B, underscoring the potential anti-AD efficacy of these DR fractions. Compound **2** exhibited excellent AChE- and BACE1-inhibitory activity, with IC_50_ values of 14.77 ± 0.07 and 44.98 ± 0.48 μM, respectively. Compounds **7** and **8** demonstrated outstanding inhibitory effects on AD-related enzymes, with IC_50_ values < 50 μM. Specifically, compound **8** showed high inhibitory activities against BACE1 and MAO-B, with IC50 values of 13.66 ± 0.14 and 2.64 ± 0.016 μM, respectively. Compounds **2**, **6**, **7**, **8**, **9**, and **10** reduced the expression levels of sAPPβ and β-secretase by 30%. Additionally, compounds **2** and **8** reduced sAPPβ and β-secretase expression levels by >50% at a concentration of 1.0 μM. Compounds **6** and 7 significantly reduced Aβ aggregates and effectively disassembled preformed Aβ aggregates. Compound **6** decreased Aβ aggregation by approximately 40% at a 1.0 μM concentration, while compound **7** reduced aggregation by 80% and 55% at 1.0 μM and 0.5 μM concentrations, respectively. Compound **6** degraded approximately 45% of preformed Aβ aggregates, whereas compound **7** degraded 80% and 60% at 1.0 μM and 0.5 μM concentrations, respectively.

Therefore, phenolic compounds from DR extract are a promising natural treatment for AD, highlighting the contribution of specific compounds to its efficacy. Particularly, this was the first study to confirm the excellent MAO-B-inhibitory activity of compound **8** and to re-prove its efficacy in inhibiting Aβ formation. It was also the first study to document the anti-AD efficacy of compounds **6** and **7**, especially regarding their ability to disaggregate Aβ aggregates. This study provides scientific data on the anti-AD effect of DR and suggests the possibility of developing new treatments based on DR.

## Figures and Tables

**Table 1:** Inhibitory activity of DR extracts and fractions on cholinesterase (AChE and BChE), BACE1, and MAO-B.

Sample	IC_50_^1^ (μg/mL)			
	AChE	BChE	BACE1	MAO-B
Ext.	>10,000	>10,000	207.07 ± 5.14	>10,000
Dichloromethane Fr.	>10,000	>10,000	>10,000	>10,000
Ethyl acetate Fr.	618.33 ± 1.75	391.70 ± 3.12	85.36 ± 3.24	75.22 ± 1.02
*n*-Butanol Fr.	607.73 ± 2.07	439.14 ± 3.12	97.82 ± 4.11	869.90 ± 25.46
Water Fr.	>10,000	>10,000	>10,000	>10,000
Berberine ^2^	0.04 ± 0.001	0.96 ± 0.08		
Quercetin ^3^			5.25 ± 0.18	
Selegiline ^4^				0.76 ± 0.03

Data are expressed as the mean ± standard deviation (*n* = 3). ^1^ IC_50_ calculated from the least-squares regression line of the logarithmic concentrations plotted against the residual activity. ^2^ Berberine was used as a positive control for cholinesterase-inhibitory activity. ^3^ Quercetin was used as a positive control for BACE1-inhibitory activity. ^4^ Selegiline was employed as a positive control for MAO-B-inhibitory activity.

**Table 2:** Inhibitory activity of ten DR compounds on cholinesterase (AChE and BChE), BACE1, and MAO-B.

Compound	IC_50_^1^ (μM)			
	AChE	BChE	BACE1	MAO-B
1	33.39 ± 0.42	53.78 ± 1.15	>1000	>1000
2	14.77 ± 0.07	488 ± 10.11	44.98 ± 0.48	401.31 ± 2.66
3	ND ^5^	ND ^5^	234.61 ± 6.86	154.94 ± 7.18
4	ND ^5^	30.63 ± 0.38	147.43 ± 0.74	600.93 ± 3.72
5	>1000	38.76 ± 0.71	>1000	ND ^5^
6	353.43 ± 0.32	32.61 ± 5.66	83.27 ± 0.33	109.19 ± 0.08
7	47.33 ± 0.22	28.83 ± 0.31	39.11 ± 0.17	65.84 ± 3.72
8	31.65 ± 0.14	30.04 ± 0.23	13.66 ± 0.14	2.64 ± 0.016
9	124.32 ± 35.33	251.96 ± 12.27	64.95 ± 0.17	51.26 ± 0.14
10	57.70 ± 5.96	331.77 ± 0.14	55.72 ± 0.17	98.77 ± 0.14
Berberine ^2^	0.31 ± 0.01	1.82 ± 0.33		
Quercetin ^3^			9.72 ± 3.72	
Selegiline ^4^				0.04 ± 0.001

Data are expressed as the mean ± standard deviation (*n* = 3). ^1^ IC_50_ calculated from the least-squares regression line of the logarithmic concentrations plotted against the residual activity; ^2^ berberine was used as a positive control for cholinesterase-inhibitory activity; ^3^ quercetin was used as a positive control for BACE1-inhibitory activity; ^4^ selegiline was employed as a positive control for the inhibitory activity for MAO-B; ^5^ ND represents no detection using test concentration.

## Data Availability

All data are contained within the article and Supplementary Materials.

## Supplementary Materials

The following supporting information can be downloaded at: https://www.mdpi.com/article/10.3390/ph17081061/s1, Figure S1: Whole Western Blot Analysis of APP-CHO Cells Treated with Six Phenolic Compounds Isolated from DR; Figure S2: Effects of Six Phenolic Compounds Isolated from DR on the Viability of APP-CHO Cells.
