# Supplementary material for: Neuroprotective Effects of Phenolic Constituents from Drynariae Rhizoma
Source: Pharmaceuticals (Basel). 2024 Aug 13;17(8):1061. doi: 10.3390/ph17081061 (PMC11358882; doi:10.3390/ph17081061)

Figure S1: Whole Western Blot Analysis of APP-CHO Cells Treated with Six Phenolic Compounds Isolated from DR. (A) shows the results for (a) sAPPβ, (b) β-secretase, and (c) α-tubulin for compounds C2 and C6. (B) shows the results for (a) sAPPβ, (b) β-secretase, and (c) α-tubulin for compounds C7 and C8. (C) shows the results for (a) sAPPβ, (b) β-secretase, and (c) α-tubulin for compounds C9 and C10.

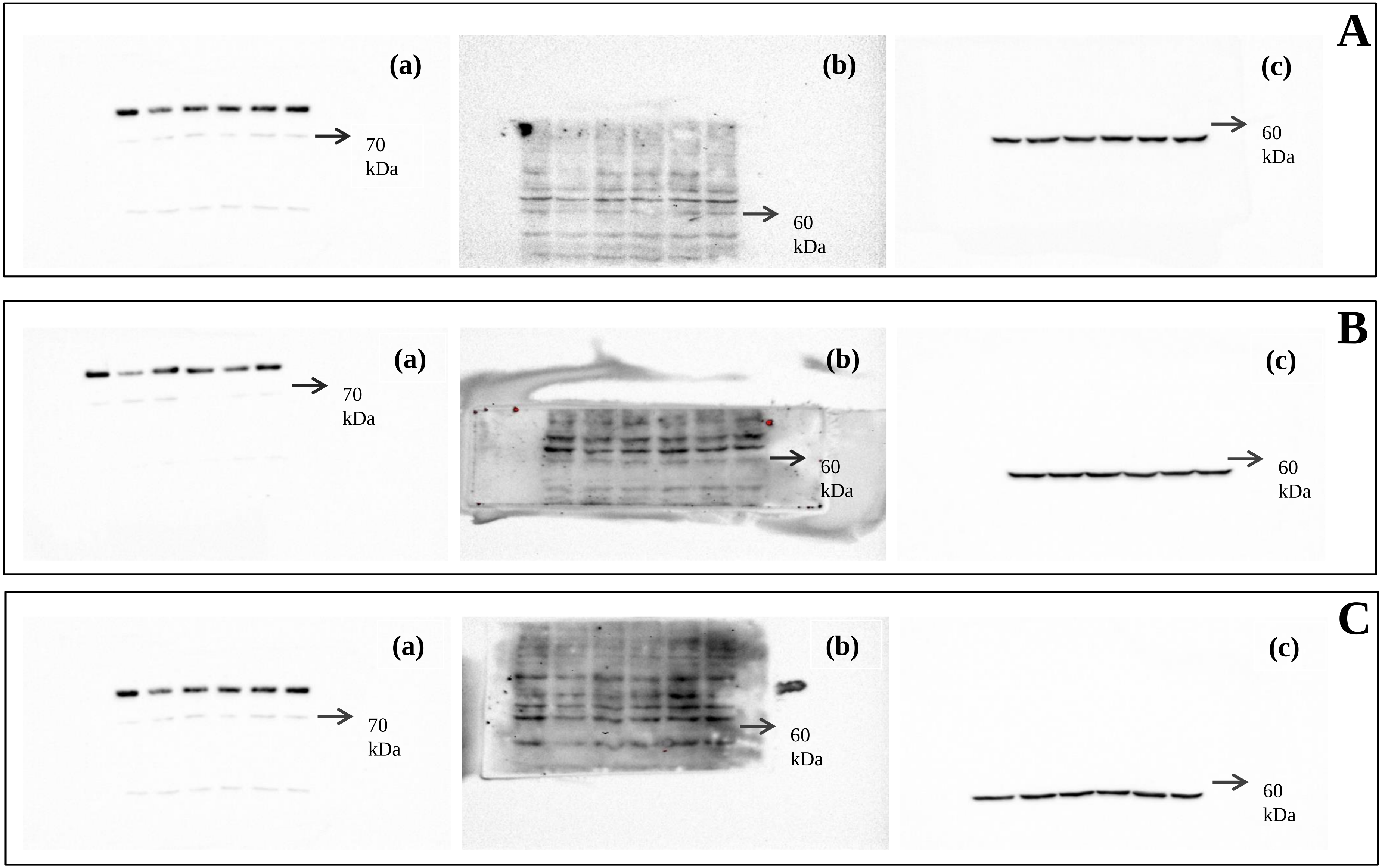

Figure S2: Effects of Six Phenolic Compounds Isolated from DR on the Viability of APP-CHO Cells.

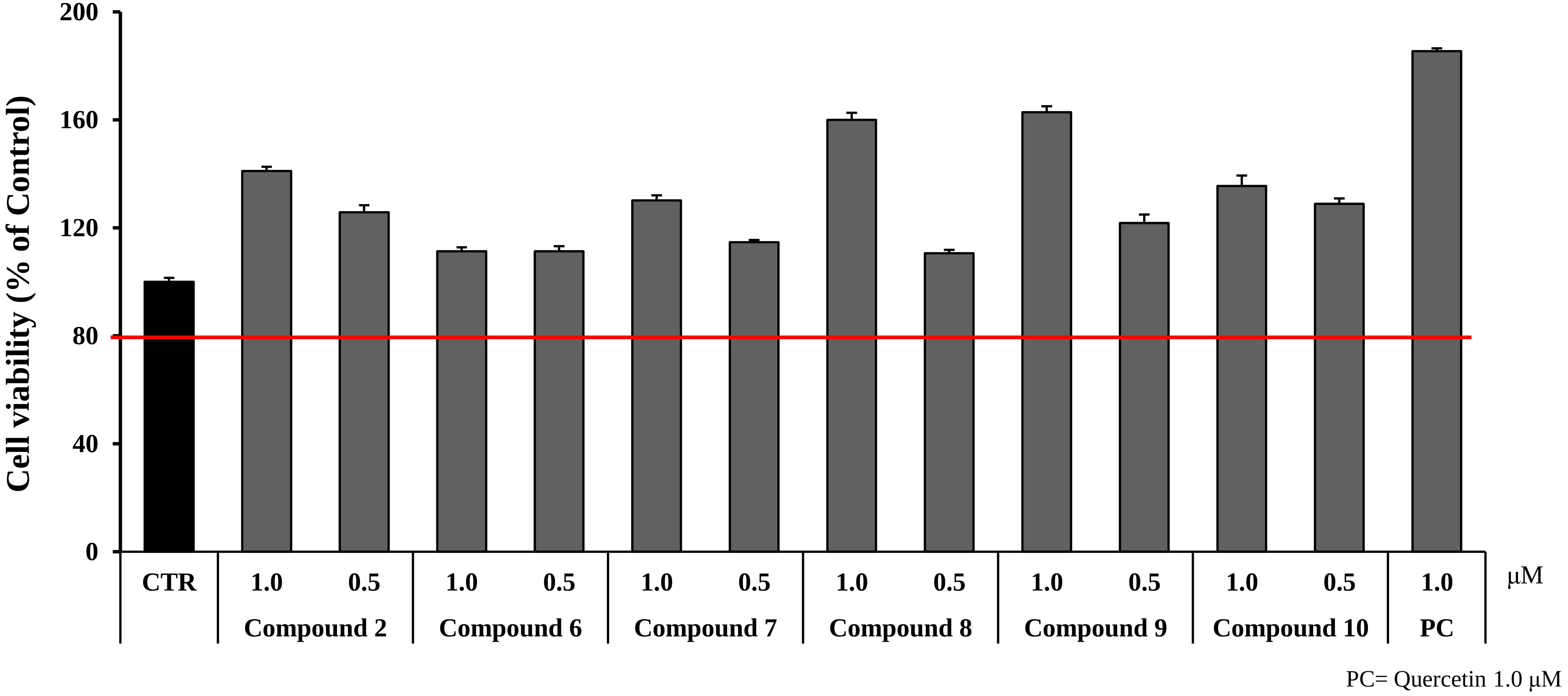

Supplement: Supplementary file 1 [file pharmaceuticals-17-01061-s001.zip › pharmaceuticals_ Supplementary Materials.pdf]
